# Effects of Roasted *Schisandra Chinensis* (Turcz.) Baill and *Lycium Chinense* Mill. and Their Combinational Extracts on Antioxidant and Anti-Inflammatory Activities in RAW 264.7 Cells and in Alcohol-Induced Liver Damage Mice Model

**DOI:** 10.1155/2021/6633886

**Published:** 2021-09-17

**Authors:** Ha-Rim Kim, Sol Kim, Seon-Young Kim

**Affiliations:** Jeonju AgroBio-Materials Institute, 54810, Wonjangdong-gil 111-27, Deokjin-gu, Jeonju-si, Jeollabuk-do, Republic of Korea

## Abstract

*Schisandra chinensis* (Turcz.) Baill (SC) and *Lycium chinense* Mill. (LC) are widely distributed in Asia, where the fruit has traditionally been used for medicinal herbs. We previously reported that the roasting process improved the antioxidant and their hangover relieving effects. In this study, we assessed the antioxidant and anti-inflammatory effects of water extract of SC, LC, and a mass ratio 1 : 1 mixture (SL), after roasting in RAW264.7 macrophage cells stimulated with lipopolysaccharide (LPS). Roasted SL (RSL) extracts showed greater enhancement potential than the others, based on the inhibition of NO (nitric oxide) and intracellular reactive oxygen species (ROS) production in RAW264.7 cells. RSL also significantly decreased the proinflammatory markers (e.g., iNOS, COX-2, TNF-*α*, and IL-1*β*) and NAD(P)H oxidase (NOX) signaling proteins (i.e., NOX (−1, −2, and −4), p22^phox^, p47^phox^, and p67^phox^). The inflammatory cytokine, tumor necrosis factor-alpha, interferon-1 beta levels, NF-kB, and mitogen-activated kinase activations were also significantly inhibited by RSL treatment. Based on the results of cellular levels, we compared the promotion effects of RSL extract on liver injury mediated by alcohol-induced inflammation and oxidative stress in mice. Mice were fed a Lieber–DeCarli regular liquid alcohol diet with or without SL and RSL extracts for six weeks. Alcohol intake caused liver injury, evidenced by an increase in serum alanine aminotransferase and aspartate aminotransferase activities. Consistent with the results in cell levels, RSL treatment remarkably downregulated ROS and inflammatory factors, as well as their signaling molecules, in serum and tissues. These results suggest that the roasting of SC and LC could potentially elevate the inhibition effect on alcohol-induced inflammation and oxidative stress and consequently prevent alcoholic liver damage. Also, the combination of SC and LC may provide a more synergistic effect than either alone.

## 1. Introduction

ALD is one of the most important health problems in the world and also a major cause of death from liver disease [1]. Ethanol induces the oxidation of liver cytochrome P450-2E1 (CYP2E1) enzymes, and oxidation of CYP2E1 leads to the overproduction of reactive oxygen species (ROS), such as hydroxyl radicals, hydrogen peroxide, and peroxide anions. These metabolites inhibit or deplete the endogenous enzymes and nonenzymatic agent, to induce oxidative stress, which may lead to apoptosis of liver cells and liver lipid peroxidation. NAD(P)H oxidases (NOX) are well-known main sources of prooxidants [2, 3]. It was also shown recently that mice deficient in NOX (p47phox knockout mice) were completely protected against alcohol-induced liver disease (ALD) in the enteral ethanol feeding model [3]. The inducible forms of NOS (iNOS) and cyclooxygenase- (COX-) 2 are another inducible enzyme that is involved in the inflammatory process and a rate-limiting enzyme [4]. A number of studies have reported that the iNOS produces NO by inflammatory stimuli, such as lipopolysaccharide (LPS), and subsequently activates COX-2, resulting in the increased release of proinflammatory prostaglandins [5]. In ALD, enhanced ROS induces the activation of nuclear factor-kappa B (NF-*κ*B) and mitogen-activated kinase (MAPK) family, which are extracellular signal-related kinases (ERKs), c-Jun N-terminal kinase (JNK), and p38 MAPK, which further evokes proinflammatory cytokines, such as tumor necrosis factor -(TNF-) *α* and interleukin- (IL-) 1*β* [6]. Chronic activation of the MAPK has been shown to increase the incidence of inflammatory diseases, due to the induction of iNOS expression. Therefore, targeting MAPK and NF-*κ*B signaling pathways has important implications as they are directly involved in the synthesis of proinflammatory mediators in activated macrophages [7–9]. *Schisandra chinensis* (Turcz.) Baill (SC) and *Lycium chinense* Mill. (LC) have been well reported in traditional Chinese medicine. SC has been reported to use antioxidant [10, 11], anti-inflammatory, antidiabetic, and nonalcoholic fatty liver disease improvement [11]. LC has also been reported to have various biological effects, including antioxidant, anti-inflammation [12], neuroprotective [13], and hepatoprotective activities [14, 15]. It has been found that thermal processing, such as roasting, homogenization, and pasteurization, increases the nutritional value of plant products and increases the release of antioxidant compounds from the cellular structure [16]. We previously reported that the antioxidant and their hangover relieving effects of SC and LC were improved after roasting [17]. In this study, the effects of water extracts of SC, LC, and mass ratio 1 : 1 mixture (SL) after roasting on LPS-induced inflammation in RAW264.7 were compared with those before roasting. As a result, the group treated with roasted SL (RSL) more effectively inhibited the expression of iNOS, COX-2, TNF-*α*, and IL-1*β* by LPS in RAW264.7 cells compared to the group treated with unroasted SL. We further investigated the effect of RSL on the NOX, NF-*κ*B, and MAPK signaling pathways, to clarify its inhibitory mechanism. The hepatoprotective and anti-inflammatory effects of RSL were also examined in an ALD mice model, using the Lieber–DeCarli liquid diet. These results provide evidence that RSL may be a potential anti-inflammatory supplement in alcoholic liver damage.

## 2. Materials and Methods

### 2.1. Materials and Antibodies

Dulbecco's modified Eagle's medium (DMEM), phosphate-buffered saline (PBS), fetal bovine serum (FBS), and antibiotics (amphotericin B, penicillin, and streptomycin) were purchased from Invitrogen (Carlsbad, CA, USA). The mouse TNF-*α*, IL-1*β* enzyme-linked immunosorbent assay (ELISA) kit was obtained from the R&D System (Abingdon, UK). The primary and secondary antibodies used in Western blot analyses were purchased from Cell Signaling Technology Inc. (Beverly, MA, USA). All other chemicals were obtained from Sigma (St Louis, MO, USA).

### 2.2. Preparation of Samples

Samples were prepared in accordance with a previous method [18], with a slight modification*. Schisandra chinensis* (Turcz.) Baill (SC) and *Lycium chinense* Mill. (LC) were obtained from a local herbal market (Seoul, Korea). Botanical identification was performed by Prof. Hong-Jun Kim (College of Oriental Medicine, Woosuk University, Jeonju, Korea). Dried SC and LC were roasted at 220°C for 9 min in a 30% aqueous ethanol using a CBR-101A roaster (Genesis, Korea). Each sample (5 g) was extracted twice in 5 mL of distilled water for 3 h at 95°C. The extracts were filtered and evaporated using a rotary evaporator (EYELA, Japan) at (40–50)°C. Finally, the extracts were lyophilized in a freeze drier (Il Shin, Korea). Samples were prepared at 100 mg/mL concentration in dimethyl sulfoxide for further study.

### 2.3. Quantification of Phytochemicals

The quality evaluation of SC and LC was performed with three reference compounds of schisandrin, gomisin A, gomisin and N, and betaine, respectively, by high-performance liquid chromatography (HPLC), equipped with a quaternary pump, an auto-sampler, a vacuum degasser, an automatic thermostatic column compartment, and UV detector. The phytochemicals from the SC and RSC were analyzed using a HPLC-MWD (Agilent 1200 series, Agilent Technologies, Palo Alto, CA, USA). An Aegispak C18 (150 mm × 4.6 mm, 3 *μ*m) column equipped with a C18 security guard (4 mm × 4.6 mm) was used at a temperature of 35°C, a flow rate of 0.5 mL/min, and detection at 254 nm. A binary gradient system with eluent (A) 0.1% formic acid in water, eluent (B) acetonitrile, and the following gradient was used: 10% B (0–10 min), 25% B (10 min), 60% B (15 min), 70% B (22–29 min), 90% B (29–33 min), 60% B (35 min), 40% B (40 min), and 10% B (45 min). For quantification of betaine in LC, the standard and LC or RLC extracts were derivatized based on a previously described method using 4-bromophenacyl bromide [19]. The derivatized LC and RLC were analyzed using a HPLC-DAD (Agilent 1200 series, Agilent Technologies). A Luna SCX C18 100 Å (150 mm × 3 mm, 5 *μ*m) column equipped with a C18 security guard (4 mm × 4.6 mm) was used at a temperature of 50°C, a flow rate of 2.0 mL/min, and detection at 254 nm. The eluent for betaine consisted of isocratic condition at 90% acetonitrile and 10% water containing 22 mM choline chloride. The results were calculated on the basis of mg/g of dry weight material.

### 2.4. Cell Culture and Cell Viability Assay

Raw 264.7 macrophages (TIB-71) were obtained from the American Type Culture Collection (ATCC, Manassas, VA, USA) and cultured in Dulbecco's modified eagle's medium (DMEM) containing 10% fetal bovine serum (FBS) and penicillin-streptomycin (100 units/mL and 100 *μ*g/mL) and maintained in 5% CO_2_ atmosphere at 37°C. The cell viability was measured by MTT assay. Cells (1 × 10^4^ cells/well) were seeded in 96-well plates and incubated with different doses of (50, 100, 250, or 500) *μ*g/mL of raw or roasted (R) SC, LC, or SL. After 24 h, 10 *μ*L MTT solution (5 mg/mL) was added to each well, and the cells were further incubated for 4 h at 37°C. The supernatant was removed, and the formazan was resolved with 100 *μ*L of DMSO. Absorbance was measured by a microplate reader (Multiskan Go, Thermo Scientific, Waltham, MA, USA) at 590 nm. The values of control were considered 100% viable.

### 2.5. NO Assay

Nitrite, which accumulated in culture supernatants, was measured using a method based on the Griess reaction as an indicator of NO production [20]. Cells (5 × 10^5^ cells/well) were incubated in 6-well plates and treated with raw or roasted SC, LC, or SL extract (500 *μ*g/mL) and then stimulated with LPS (1 *μ*g/mL) for 18 h. The culture medium was mixed with Griess reagent (equal volumes of 1% sulfanilamide in 5% phosphoric acid and 0.1% N-(1-naphthyl) ethylenediamine dihydrochloride) and further incubated for 20 min at room temperature (RT). The concentration of nitrite was determined using the sodium nitrite (NaNO_2_) standard curve at 540 nm with a microplate reader.

### 2.6. Measurement of ROS

Generation of intracellular ROS was measured with the FluoroProbe DCF-DA. Different doses of raw or roasted SC, LC, or SL extract were treated and incubated with 10 *μ*M DCF-DA for 60 min at 37°C. The cells were digested with 1 N NaOH, followed by a quantitative analysis of fluorescence intensity using a fluorescence microplate reader (excitation 488 nm, emission 513 nm).

### 2.7. Determination of the Anti-Inflammatory Activity

RAW 264.7 cells (5 × 10^5^ cells/well) were seeded in six-well plates with raw or roasted SC, LC, or SL extract (500 *μ*g/mL) and then stimulated with LPS (1 *μ*g/mL) for 18 h at 37°C in a 5% CO_2_ atmosphere. The culture medium was then harvested, and the cytokine production levels in the supernatants were measured using enzyme-linked immunosorbent assay (ELISA) kits, according to the manufacturer's instructions (R&D System, Abingdon, UK). The absorbance was measured using an ELISA reader at 450 nm (Multiskan Go, Thermo Scientific, Waltham, MA, USA). Mouse TNF-*α* and IL-1*β* production levels were used to assess mouse liver homogenates.

### 2.8. Animals

C57BL/6J (five-week-old, male) mice were obtained from Damul Science (Daejeon, Korea). Mice were randomly divided into five groups and housed under standard conditions at 22 ± 2°C and 55 ± 5% humidity and 12 h light/dark cycles. Mice were allowed to adapt to the conditions for one week before the initiation of experiments and were provided water and food *ad libitum*. The experimental protocol for this study was reviewed and approved by the Animal Care Committee of the Jeonju AgroBio-Materials Institute, and the committee guidelines (JAMI IACUC 2018004) were strictly followed.

### 2.9. Chronic Alcoholic-Fed Liver Injury Model

For induction of chronic alcoholic liver disease development, mice were fed a Lieber–DeCarli regular liquid diet (Dyets, Inc., Bethlehem, PA, USA), which allowed *ad libitum* access to the liquid diet. In contrast, mice of normal and negative control groups were fed a Lieber–DeCarli regular liquid diet control. Each sample was orally administered to the experimental group for 6 weeks, and the normal and negative control groups were administered the same volume of vehicle. Mice were weighed once a week on the same day and time. At the end of the experiment, blood was collected from the abdominal vena cava of sacrificed mice, and liver tissue was excised and weighed for histopathological studies. The liver index was determined as the ratio of liver weight to body weight. The liver tissue was lysed in RIPA buffer (10 mM Tris-HCl, pH 7.5, 0.1% NP-40, 0.5% sodium deoxycholate, 0.1% SDS, 1 mM sodium orthovanadate, 120 mM sodium chloride, 1 mM phenylmethylsulfonyl fluoride, 10 *μ*g/mL leupeptin, and 1 *μ*g/mL aprotinin) with a homogenizer. After centrifugation at 12,000 rpm for 10 min, the liver homogenates were collected. Serum was obtained by centrifugation at 3,000 rpm for 15 min followed by centrifugation at 12,000 rpm for 10 min. Serum and liver tissue homogenates were aliquoted and stored at −20°C for subsequent experiments. The serum activities of alanine aminotransferase (ALT) and aspartate aminotransferase (AST) levels were determined using a commercially available kit (BioVision Inc., CA, USA), according to the manufacturer's instructions.

### 2.10. Determination of the Antioxidant Activity

The hepatic oxidative stress level was assessed by measuring superoxide dismutase (SOD) activities and glutathione (GSH) levels. SOD activities were measured using a SOD assay kit (Sigma, MO, USA), according to the manufacturer's instructions. Briefly, 20 *μ*L of serum and tissue homogenate was mixed with 200 *μ*L of WST-1 (2-(4-iodophenyl)-3-(4-nitrophenyl)-5-(2, 4-disulfophenyl)-2H-tetrazolium, monosodium salt) working solution in a 96-well plate. Then, 20 *μ*L of enzyme working solution was added to the mixture and incubated for 20 min at 37°C. The plate was measured at 450 nm by UV spectrophotometry (Thermo Scientific, Germany). Glutathione levels were determined using a GSH assay kit (Cayman, MI, USA). Serum and tissue homogenate added triethanolamine solution and Assay Cocktail (a mixture of MES buffer (11.25 mL), reconstituted cofactor mixture (0.45 mL), reconstituted enzyme mixture (2.1 mL), water (2.3 mL), and DTNB (0.45 mL), and total GSH in the deprotonated sample were measured using UV spectrophotometry at 412 nm.

### 2.11. Immunoblotting

Protein extraction and immunoblotting of cells and liver tissues were performed as previously described. Proteins (20 *μ*g per lane) were resolved on 8 or 10% SDS-PAGE gel and transferred to polyvinylidene difluoride (PVDF, GE Healthcare, Little Chalfont, Buckinghamshire, UK) membranes. Blots were incubated with primary antibodies (1 : 2,500 dilution of each antibody) overnight at 4°C. The blots were rinsed four times with blocking buffer and incubated with horseradish peroxidase-conjugated secondary antibodies (1 : 5,000 dilution of each antibody) for 1 h. The binding of the antibodies was visualized using an enhanced chemiluminescence (ECL) system (Bio-Rad, Munich, Germany). Protein concentrations were determined using a Bio-Rad protein assay kit, and known concentrations of bovine serum albumin (BSA) were used as the standard. All immunoreactive signals were analyzed by densitometric scanning (Amersham imager 600, GE Healthcare, Buckinghamshire, UK).

### 2.12. Histological Analysis

Mouse liver tissues were fixed in 4% paraformaldehyde for 24 h and embedded in paraffin. Serial sections (4 *μ*m) were cut, deparaffinized, hydrated, and stained with hematoxylin and eosin (H&E). All stained specimens were observed and photographed by optical microscopy (Olympus, Tokyo, Japan).

### 2.13. Statistical Analysis

Data represent the mean ± standard deviation (SD) of at least three separate experiments. All statistical analyses were determined by one-way ANOVA (one-way analysis of variance) analysis. In all statistical comparisons, *P* < 0.05 was used to indicate a statistically significant difference.

## 3. Results and Discussion

### 3.1. Analysis of Active Compounds in SC and LC after the Roasting Process

We evaluated the contents of active compounds using HPLC-DAD analysis, including schisandrin and gomisin (A and N) in SC and betaine in LC extracts, after roasting at 220°C for 9 min. The roasting conditions were adopted according to our previous studies [17, 18].  Table 1 shows that the contents of schisandrin and gomisin (A and N) were 41.89, 8.01, and 3.76 mg/g of raw SC, respectively. The content of each compound in roasted SC has increased by about 2 to 4 times compared to the raw material. The content of betaine in roasted LC also increased more than 2 times compared to the raw material (Table 1).

### 3.2. Effect of Roasted SL on LPS-Induced NO Production and ROS Generation in RAW 264.7 Cells

**Figure 1:**
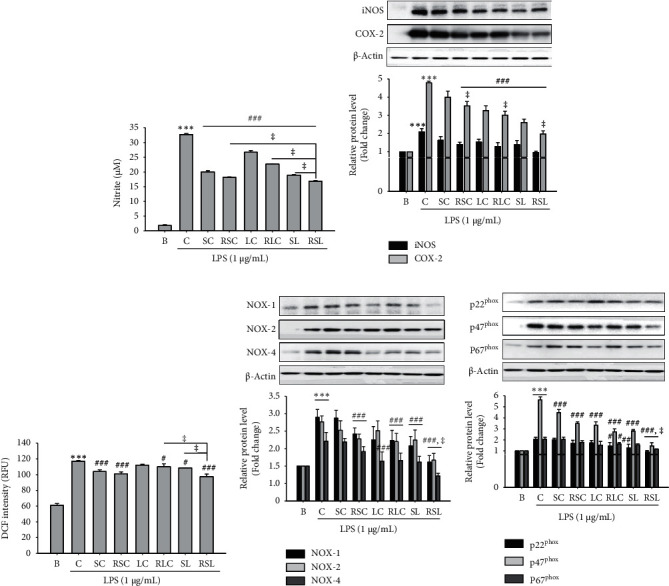
The effects of raw or roasted *Schisandra chinensis* (Turcz.) Baill (SC), *Lycium chinense* Mill. (LC), or mass-based 1 : 1 mixture, SL, on LPS-stimulated nitric oxide (NO) production, ROS generation, and the protein expression of iNOX, COX-2, and NADPH oxidases (NOXs) in RAW264.7 macrophages. RAW264.7 cells were pretreated with raw or roasted (R) SC, LC, or SL (500 *μ*g/mL) for 1 h and then incubated with LPS (1 *μ*g/mL) for 18 h. (a–e) The culture media were collected for NO production, cell lysates were prepared for ROS generation, and iNOS, COX-2, NOX (−1, −2, and −4), and p22^phox^, p47^phox^, and p67^phox^ levels were analyzed by immunoblotting. The densitometry data represented are shown as the relative density of protein bands normalized to *β*-actin level.  ^*∗∗∗*^*P* < 0.001 vs. blank B; ^#^*P* < 0.05, ^##^*P* < 0.01, ^###^*P* < 0.001 vs. control (C: LPS treatment group). ^ǂ^*P* < 0.05 vs. RSC, RLC, and SL. Values are the mean ± SEM of three independent experiments.

Firstly, the effects of raw or roasted SC, LC, or mass ratio 1 : 1 mixture (SL) extracts on the cell cytotoxicity were investigated. As shown in Supplementary Figure 1, none of the samples were toxic to RAW264.7 cells at the treatment concentration range of 50–500 *μ*g/mL. Based on the toxicity results, subsequent analysis of the effect of each sample was compared at a concentration of 500 *μ*g/mL. We investigated the effect of roasted SC, LC, and SL and their raw materials on LPS-induced NO and ROS production and related signal protein expression in RAW264.7 cells. LPS-induced NO production was inhibited by SC, RSC, LC, RSL, SL, and RSL extracts treatment at 500 *μ*g/mL (Figure 1(a)). Compared to the group treated with RSC, RLC, and their raw materials, the roasted SL-treated group significantly suppressed LPS-induced NO production in RAW264.7 cells (*P* < 0.05) (Figure 1(a)). The expression levels of iNOS and COX-2 were significantly upregulated in the LPS-treated control (C) group by 2.10- and 4.79-fold (*P* < 0.001), compared with the blank (B) group (Figure 1(b)). Treatment with raw or roasted SC, LC, or SL showed a potent effect on the reduction of LPS-stimulated iNOS and COX-2 protein expression levels in RAW264.7 cells (Figure 1(b)). We further analyzed whether RSL regulates intracellular ROS levels in RAW264.7 cells. As a result, we found that pretreatment of cells with roasted SC, LC, or SL reduced LPS-induced ROS (Figure 1(c)). NADPH oxidase (NOX) has been reported as the main factor in LPS-mediated ROS generation in macrophages [21]. We tested the effects of each extract on the expression of NOX (−1, −2, and −4), p22^phox^, p47^phox^, and p67^phox^ in RAW264.7 cells. As shown in Figures 1(d) and 1(e), the protein levels of NOX (−1, −2, and −4), p22^phox^, p47^phox^, and p67^phox^ were obviously increased in the LPS-treated group by 2.39-, 3.03-, 1.69-, 2.06-, 5.64-, and 1.84-fold (*P* < 0.001). RSL treatment showed the most effective results in suppressing LPS-mediated ROS generation and NOX signaling protein expression in RAW264.7 cells (Figures 1(c)–1(e)).

### 3.3. RSL Inhibits Proinflammatory Cytokines Production and MAPK and NF-kB Activation in LPS-Stimulated RAW 264.7 Cells

**Figure 2:**
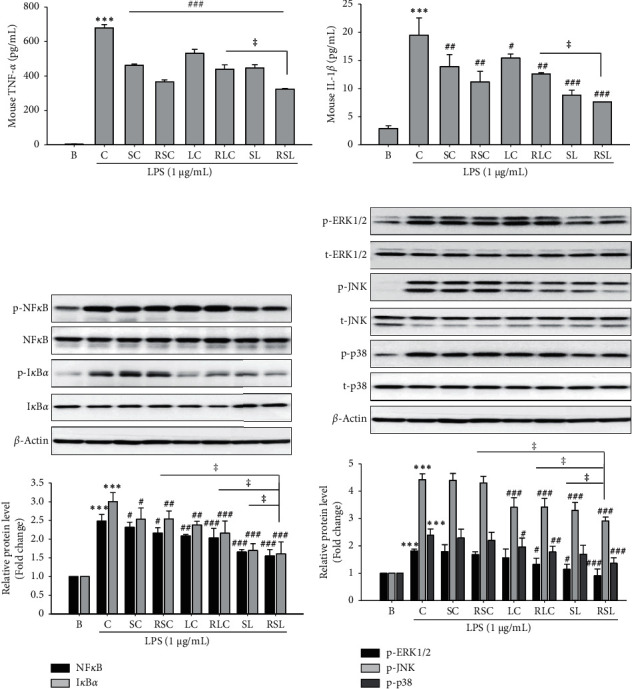
The effect of raw or roasted SC, LC, or SL on the production of proinflammatory factors and NF-*κ*B and MAPKs signaling in LPS-induced RAW264.7 cells. Cells were seeded in 6-well plates and the indicated samples were pretreated at 500 *μ*g/mL for 1 h and then stimulated with LPS (1 *μ*g/mL) for 18 h. The concentrations of (a) TNF-*α* and (b) IL-1*β* in the culture medium were measured by ELISA. Cell lysates were analyzed by western blotting to determine the protein levels. (c) Phospho-NF-*κ*B and -I*κ*B levels. (d) Phospho-ERK1/2, -JNK, and -p38 levels. The total NF-*κ*B, I*κ*B, ERK1/2, JNK, or p38 staining is shown as a loading control, respectively. The quantitative results are depicted. The densitometry data represented are shown as the relative density of protein bands normalized to *β*-actin level.  ^*∗∗∗*^*P* < 0.001 vs. B; ^#^*P* < 0.05, ^##^*P* < 0.01, ^###^*P* < 0.001 vs. C ^ǂ^*P* < 0.05 vs. RSC, RLC, or SL. Values are the mean ± SEM of three independent experiments.

We further studied the effects of RSL extracts on inflammatory cytokine production and signaling molecules, including NF-*κ*B and MAPK. Among the various cytokines, TNF-*α* and IL-1*β* have emerged as critical factors in inflammatory diseases [20]. To determine whether RSL affects the release of cytokines in LPS-stimulated Raw264.7 cells, we analyzed TNF-*α* and IL-1*β* levels. LPS significantly increased intracellular TNF-*α* and IL-1*β* levels in RAW264.7 cells, which were effectively inhibited by RSC, RLC, or RSC as well as their raw materials (Figures 2(a) and 2(b)). It is well known that NF-kB is an important transcription factor for the activation of iNOS, COX-2, TNF-*α*, and IL-1*β* in inflammatory pathogenesis [22]. Thus, the suppression of NF-*κ*B may be an effective therapeutic strategy for preventing inflammatory processes and diseases [8, 9]. As shown in Figure 2(c), raw or roasted SC, LC, and SL significantly inhibited LPS-induced NF-kB and IkBa phosphorylation in RAW 264.7 cells.

MAPK signaling pathways were also directly involved in the modulation of proinflammatory modulators in activated macrophages [7, 9]. In the MAPK family, ERKs, JNKs, and p38 are the most important components. In the present study, we tested the effect of raw or roasted SC, LC, or SL extracts on the LPS-induced phosphorylation of ERK1/2, JNK, and p38 in RAW264.7 cells. The raw and roasted LC and SL extracts showed a remarkable reducing effect on the LPS-induced phosphorylation of ERK1/2, JNK, and p38 in RAW264.7 cells (Figure 2(d)). On the other hand, SC extract showed no significant effect (Figure 2(d)). Similar to the above data, RSL showed the most potent effect on NF-*κ*B and MAPK.

### 3.4. Effect of Roasted SL on ALD-Associated Liver Damage

**Figure 3:**
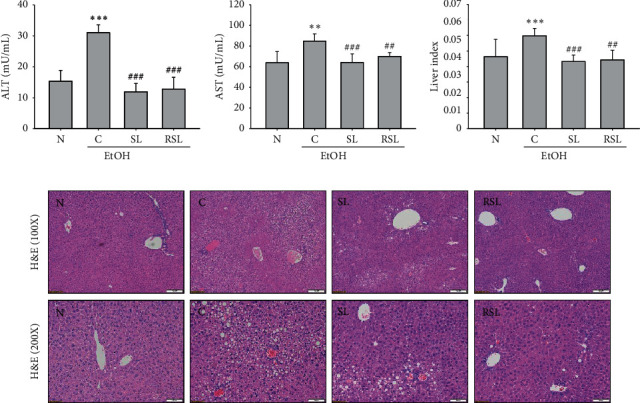
Effect of SL or RSL in alcohol-induced hepatic damage. Effects of SL or RSL on alcohol-induced (a) ALT, (b) AST, and (c) liver index. (d) Histological evaluation of liver in ethanol-fed mice by hematoxylin and eosin staining (×100 and ×200).  ^*∗∗*^*P* < 0.01,  ^*∗∗∗*^*P* < 0.001 vs. N; ^##^*P* < 0.01, ^###^*P* < 0.001 vs. C. N: normal group; C: ethanol-fed control group; SL: ethanol-fed group with SL treatment; RSL: ethanol-fed group with RSL. Values represent the mean ± SEM for 6 mice per group.

The above results showed that RSL had the most significant effect on the LPS-induced inflammatory response in RAW264.7 cells. To validate the anti-inflammatory efficacy of RSL, we further evaluated its effect in the Lieber–DeCarli liquid diet-induced ALD mouse model. The increased activity of AST and ALT in serum indirectly reflects ethanol-induced liver damage. As shown in Figures 3(a) and 3(b), serum ALT and AST levels were significantly increased in the ethanol-administered group (*P* < 0.05). On the other hand, the levels of ALT and AST were significantly reduced in the group administered with SL and RSL (Figures 3(a) and 3(b)). In addition, the liver-to-body ratio of mice in the ethanol-fed group was significantly increased, which was effectively improved by administration with SL and RSL (Figure 3(c)). To further evaluate the hepatoprotective effects of SL and RSL, we observed changes in liver tissue after H&E staining. Compared with the normal group, liver tissue from ethanol-fed mice had an incomplete structure of most hepatic lobules; lipid droplet accumulation and inflammatory cell infiltration were found (Figure 3(d)). In contrast, SL or RSL administration significantly inhibited lipid vacuole and inflammatory cell infiltration (Figure 3(d)). These results suggest that SL and RSL can protect against abnormal lipid accumulation and damage to liver tissue, which is exacerbated by ALD.

### 3.5. Effect of SL and RSL on Alcohol-Induced Oxidative Stress

**Figure 4:**
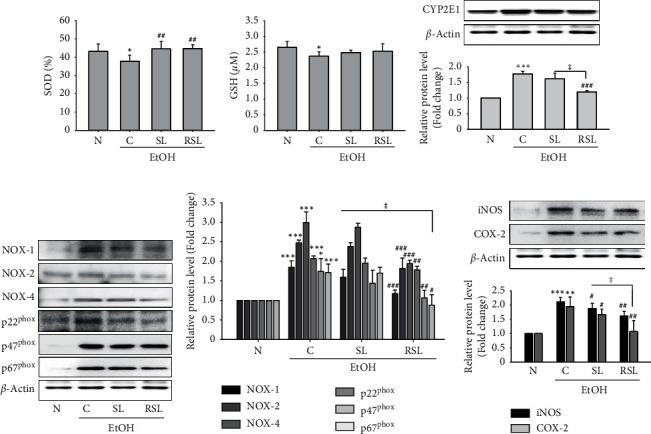
SL or RSL inhibited oxidative stress in alcohol-fed mice livers. The levels of (a) SOD and (b) GSH in liver lysates. The protein expression levels of (c) CYP2E1, (d) NOXs, including NOX (−1, −2, and −4), p22^phox^, ph47^phox^, and p67^phox^, their (e) relative density analysis of the NOXs protein bands, and (f) iNOS and COX-2 in liver lysates from all groups were analyzed by immunoblotting. The densitometry of each band was quantified by image analysis. The band density was normalized to *β*-actin, followed by statistical analysis. ^*∗*^*P* < 0.05,  ^*∗∗*^*P* < 0.001,  ^*∗∗∗*^*P* < 0.001 vs. N; ^#^*P* < 0.05, ^##^*P* < 0.01, ^###^*P* < 0.001 vs. C. ^ǂ^*P* < 0.05 vs. SL. N: normal group; C: ethanol-fed control group; SL: ethanol-fed group with SL treatment; RSL: ethanol-fed group with RSL. Values represent the mean ± SEM for 6 mice per group.

Alcohol induces oxidative stress in the liver and reduces endogenous antioxidant capacity, leading to the accumulation of ROS and ultimately accelerating liver damage [2, 23]. Figures 4(a) and 4(b) show that the SOD and GSH contents in the liver tissue of the ethanol-fed group were significantly decreased compared to the normal group. As a result of administration of SL and RSL, the level of SOD in the liver tissue was improved compared to the C group (*P* < 0.01), but the GSH content did not show a significant difference from the C group (*P* > 0.05) (Figures 4(a) and 4(b)). In ALD, ROS is generated through activation of NOX and CYP2E1 and promotes inflammation by inducing iNOS and COX-2 activity [1, 3, 24]. Therefore, it can improve ALD by inhibiting CYP2E1, NOX, iNOS, and COX-2 activation. As shown in Figure 4(c), alcohol administration increased the protein expression of CYP2E1 in liver tissue. SL and RSL treatment suppressed CYP2E1 protein expression. Moreover, RSL had a more significant effect compared to SL (*P* < 0.05). Protein levels of NOX (−1, −2, −4), p22phox, p47phox, and p67phox in liver tissue of ethanol-fed mice were also significantly increased compared to the N group. RSL treatment improved the alcohol-induced NOXs expression, but SL treatment did not show a significant inhibitory effect (Figures 4(d) and 4(e)). We further investigated the protein expression of proinflammatory factors, iNOS and COX-2, in liver tissues of each group. As shown in Figure 4(f), the protein levels of iNOS and COX-2 were significantly increased in the liver tissues of ethanol-fed mice compared to the normal group (*P* < 0.01). Both RSL- and SL-treated groups suppressed protein expression, and the RSL-treated group had a more significant effect than the SL-treated group (*P* < 0.05).

### 3.6. Effect of SL and RSL on the Activation of Hepatic Inflammatory Cytokines, MAPK, and NF-kB

**Figure 5:**
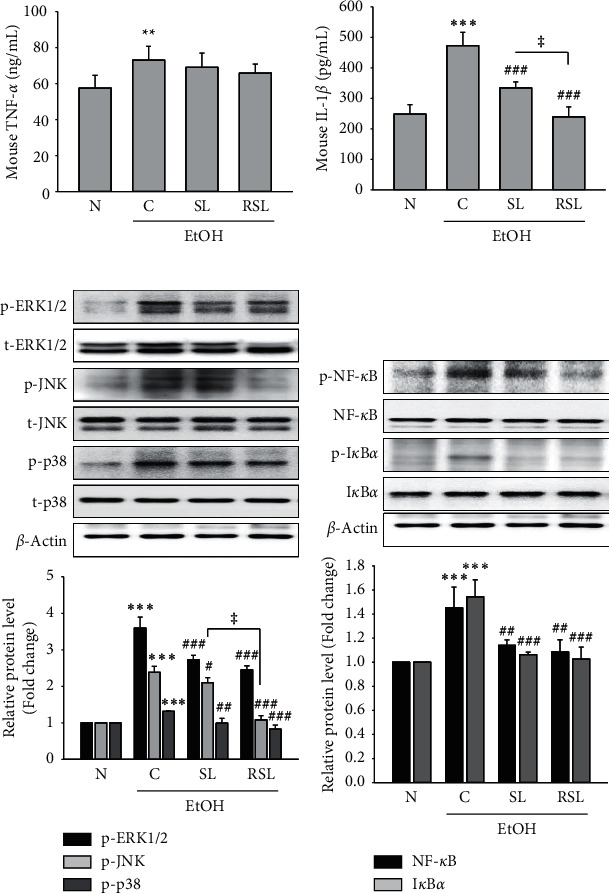
Suppression of inflammatory mediators and MAPK and NF-*κ*B signaling in alcohol-fed mice livers by SL and RSL. (a) TNF-*α* and (b) IL-1*β* levels were analyzed using ELISA kit in liver lysates. Phosphorylation of (c) ERK1/2, JNK, and p38, and (d) NF-*κ*B and I*κ*B was determined by Western blot analysis in lysed liver tissue. The total NF-*κ*B, I*κ*B, ERK1/2, JNK, or p38 staining is shown as a loading control, respectively. The quantitative results are depicted. The densitometry data represent the relative density of protein bands normalized to *β*-actin level. ^*∗*^*P* < 0.05,  ^*∗∗*^*P* < 0.001,  ^*∗∗∗*^*P* < 0.001 vs. N; ^#^*P* < 0.05, ^##^*P* < 0.01, ^###^*P* < 0.001 vs. C. ^ǂ^*P* < 0.05 vs. SL. N: normal group; C: ethanol-fed control group; SL: ethanol-fed group with SL treatment; RSL: ethanol-fed group with RSL. Values represent the mean ± SEM for 6 mice per group.

Activation of TNF-*α* and IL-1*β*, two major inflammatory mediators associated with inflammation, plays an important role in the progression of alcoholic liver injury [25]. Alcohol administration increased the levels of these inflammatory mediators, including TNF-*α* and IL-1*β*, in mice liver tissue lysates (Figures 5(a) and 5(b)). Although no statistical significance was found, TNF-*α* levels were inhibited by SL and RSL (Figure 5(a)). In contrast, IL-1*β* levels were significantly decreased by SL or RSL treatment (*P* < 0.01), as compared to that in the C group (Figure 5(b)). In a similar interpretation of the *in vitro* results, we investigated whether the anti-inflammatory effects of SL or RSL were MAPK and NF-kB in the ALD mouse model. As shown in Figures 5(c) and 5(d), ethanol administration stimulated phosphorylation of ERK1/2, -JNK, -p38, -NF-*κ*B, and -IkB*α* in the liver tissue. Both groups treated with SL and RSL significantly reduced the levels of phospho-ERK1/2, -p38, -NF-*κ*B, and -IkB*α* (Figures 5(c) and 5(d)). In the case of JNK phosphorylation, only the RSL treatment group showed a statistically significant effect.

## 4. Discussion

Chronic alcohol consumption has long been associated with progressive liver disease from steatosis to inflammation, development of hepatic cirrhosis, and the subsequent increased risk of hepatocellular carcinoma. Oxidative stress and the activation of an inflammatory cascade are identified as key factors in the pathophysiology of alcoholic liver disease (ALD) [1, 2, 26].

*Schisandra chinensis* (Turcz.) Baill (SC) and *Lycium chinense* Mill. (LC), a traditional herbal medicine, have been reported to possess antiallergic, antioxidant, anti-inflammatory, antidiabetic, and hepatoprotective effects [10, 15, 27, 28]. Thermal processing enhances the antioxidant and anti-inflammatory effects and health-relevant functionality of edible sources [16, 18]. For example, it has been shown that roasting improved the efficiency of inflammation and the inducing of Nrf2 activity in coffee [29]. Recently, we also previously reported that thermal processing increased the antioxidant and anti-inflammatory effects [17]. In this study, we found SC, LC, and their mass-based 1 : 1 mixture SL extracts have anti-inflammatory effects on RAW264.7 cells. The mixture SL was more effective than single extract; moreover, the roasted SL showed the most potent anti-inflammatory effect, both in LPS-induced RAW cells and in ALD mice model. SL or RSL treatment significantly inhibited the production of TNF-*α* and IL-1*β* and NO and the expression of iNOS and COX-2 in LPS-stimulated RAW264.7 cells (Figure 1) and alcohol-fed mice livers (Figures 4 and 5). These results suggest that roasted SL reduces the inflammation in LPS-stimulated macrophage and alcohol-fed mice livers by suppressing proinflammatory mediator activation.

NADPH oxidase (NOX) activity has been implicated as an important source of ROS during chronic ethanol exposure in rodent models [3, 24]. NOX was found in phagocytic cells involved in the innate immune response [21]. In phagocytes, NOX assembles several NOX protein components, including the catalytic subunits of the membrane-bound proteins gp91^phox^ (NOX-2) and p22^phox^; the cytosolic proteins p47^phox^, p67^phox^, and p40^phox^; and the small GTPase Rac, by bacterial exposure, and rapidly increases ROS levels. It becomes increasingly clear that NOX-dependent ROS production is not limited to phagocytes, because NOX enzymes are widely expressed and active in many different cell types, from a variety of tissues and organs [30]. NOX isoforms, including NOX (−1, −2, and −4) and NOX-derived ROS, have been implicated in regulating hepatic damage [30]. In this study, we observed that treatment with SL or RSL remarkably inhibited the LPS-induced intracellular ROS generation in RAW264.7 cells and restored antioxidant capacity in alcohol-fed liver tissue and the upregulation of NOX proteins, including NOX (−1, −2, and −4), p22^phox^, p47^phox^, and p67^phox^, in both *in vitro* and *in vivo* model (Figures 1 and 4). Furthermore, RSL showed a more potent effect, compared to SL treatment. CYP2E1 is another major cause of ROS generation [31]. CYP2E1 has been proposed as a major cause of ethanol-induced oxidative stress and liver damage because CYP2E1 can oxidize ethanol to generate reactive molecules and catalyze the production of peroxide anionic radicals and ROS. CYP2E1 knock-in mice had increased hepatic steatosis and liver damage after alcohol intake [31]. Our results showed that roasted SL significantly reduced the levels of CYP2E1 protein expression in alcohol-fed mice livers (Figure 4(c)). These results provide important evidence that the roasted SC and LC mixtures have improved antioxidant capacity in RAW264.7 macrophages and the ALD mice model.

ROS then induces lipid peroxidation and activates multiple signaling cytotoxicity pathways, including MAPKs and NF-*κ*B [7, 32]. MAPK is a serine/threonine kinase that mediates intracellular signal transduction that is associated with various cellular activities, including cell proliferation, differentiation, and survival. The mammalian MAPK family consists of extracellular signal-regulated kinase (ERK), p38, and c-Jun NH2-terminal kinase (JNK) [7]. Increased activation of this key MAPK signaling pathway after chronic ethanol feeding contributes to increased inflammatory cytokines production [7]. NF-*κ*B is a family of transcription factors and regulates the expression of a number of immune-related mediators, including iNOS and COX-2, and proinflammatory cytokines [8]. In nonstimulated cells, NF-kB is regulated by interaction with a family of regulated proteins, an inhibitor protein called I*κ*B. RSL treatment reduced the phosphorylation of MAPKs and NF-*κ*B. Activated by external stimulation, I*κ*B undergoes phosphorylation, ubiquitination, and subsequent proteolysis, causing NF-*κ*B to move into the nucleus to regulate the transcription of target genes [32]. Studies reported that the inhibition of NF-*κ*B activation can protect liver damage from experimental ALD. In agreement with this statement, we demonstrated that SL or RSL treatment inhibited ERK, JNK, and p38 MAPK phosphorylation, in response to LPS in RAW264.7 cells and alcohol-fed mice livers (Figures 2(d) and 5(c)). In addition, the current study showed that SL or RSL treatment significantly blocked NF-*κ*B and IkB phosphorylation in RAW264.7 cells and ALD mice livers (Figures 2(c) and 5(d)). Consistently, our results showed that the roasted SL revealed a more potent effect on the regulation of MAPK and NF-*κ*B signaling in both *in vitro* and *in vivo* inflammatory models.

In this study, we assessed the antioxidant and anti-inflammatory effects of raw and roasted SC, LC, or mass-based 1 : 1 mixture SL in RAW264.7 macrophages and the ALD mice model. We also evaluated the signaling molecules affected during LPS stimulation and chronic alcohol exposure in mice livers. Our results show that roasted SL is attributed to the inhibitory effect of inflammation and defenses against oxidative stress. The roasted SL can improve the protection against alcohol-induced liver damage by inhibiting oxidative stress and inflammatory mediators through the regulation of MAPK and NF-kB signaling in LPS-stimulated RAW264.7 macrophage and alcoholic liver damage.

## Figures and Tables

**Table 1:** Contents of phytochemicals in raw or roasted SC or LC extracts.

	SC			LC
	Schisandrin (mg/g)	Gomisin A (mg/g)	Gomisin N (mg/g)	Betaine (mg/g)
Raw	41.89 ± 7.44	8.01 ± 0.38	3.76 ± 0.19	15.02 ± 1.90
Roasted	167.73 ± 6.74^*∗∗∗*^	22.81 ± 1.42^*∗∗∗*^	9.08 ± 0.29^*∗∗∗*^	45.25 ± 1.80^*∗∗∗*^

Values are the mean ± SD of three independent experiments. ^*∗∗∗*^*P* < 0.001 vs. raw. SC: *Schisandra chinensis* (Turcz.) Baill; LC: *Lycium chinense* Mill. (LC).

## Data Availability

All the data generated or analyzed during this study are included in this published article.

## Supplementary Materials

Supplementary Materials Supplementary Figure 1: cytotoxicity of raw or roasted (R) SC, LC, or SL in RAW264.7 cells. Values are the mean ± SEM of three independent experiments.
