# Supplementary material for: Effects of Roasted Schisandra Chinensis (Turcz.) Baill and Lycium Chinense Mill. and Their Combinational Extracts on Antioxidant and Anti-Inflammatory Activities in RAW 264.7 Cells and in Alcohol-Induced Liver Damage Mice Model
Source: Evid Based Complement Alternat Med. 2021 Sep 17;2021:6633886. doi: 10.1155/2021/6633886 (PMC8463187; doi:10.1155/2021/6633886)
Supplement: Supplementary Materials — Supplementary Figure 1: cytotoxicity of raw or roasted (R) SC, LC, or SL in RAW264.7 cells. Values are the mean ± SEM of three independent experiments. [file 6633886.f1.docx]

**
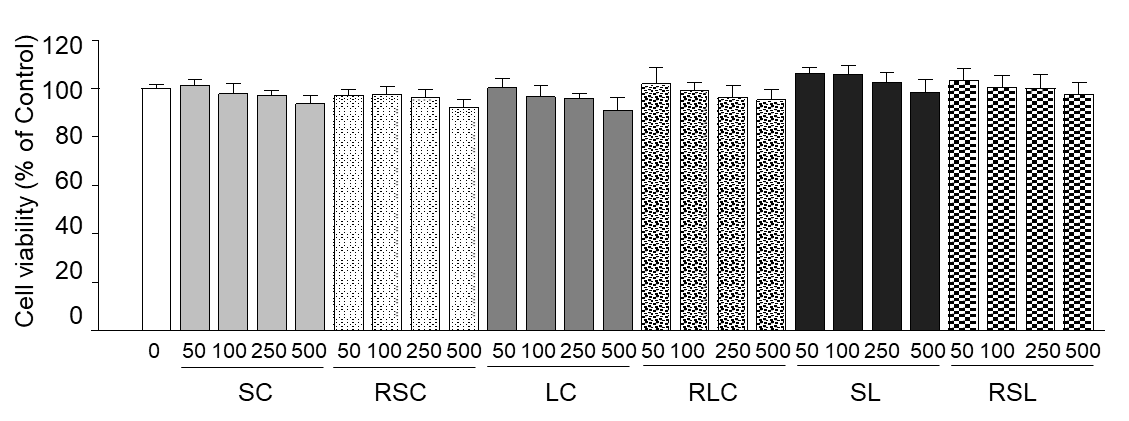
**

**Supplementracy Figure 1:** Cytotoxicity of raw or roasted (R) SC, LC, or SL in RAW264.7 cells. Values are the mean ± SEM of three independent experiments.
